# MiR-139-5p reverses CD44^+^/CD133^+^-associated multidrug resistance by downregulating *NOTCH1* in colorectal carcinoma cells

**DOI:** 10.18632/oncotarget.12611

**Published:** 2016-10-12

**Authors:** Ke Xu, Ke Shen, Xin Liang, Yueqi Li, Norio Nagao, Jiyu Li, Jianwen Liu, Peihao Yin

**Affiliations:** ^1^ Central Laboratory, Putuo Hospital and Interventional Cancer Institute of Chinese Integrative Medicine, Shanghai University of Traditional Chinese Medicine, Shanghai 200062, PR China; ^2^ State Key Laboratory of Bioreactor Engineering and Shanghai Key Laboratory of New Drug Design, School of Pharmacy, East China University of Science and Technology, Shanghai, 200237, PR China; ^3^ Department of Life and Environmental Sciences, Prefectural University of Hiroshima, Shobara, 727-0023, Japan; ^4^ Department of general surgery, Shanghai Tenth People's Hospital, Tongji University School of Medicine, Shanghai, 200072, PR China

**Keywords:** colorectal cancer, miR-139-5p, NOTCH1, drug resistance

## Abstract

MiRNAs may promote or inhibit tumor recurrence and drug resistance. MiR-139-5p is reportedly downregulated in colorectal cancer patient samples, but it is unknown whether and how miR-139-5p regulates drug resistance. Cancer stem cells (CSCs) are postulated to be important promoters of multiple drug resistance (MDR). In this study, we established a MDR cell model which strongly expressed the CSC-associated biomarkers CD44 and CD133. MiR-139-5p expression was reduced in MDR cell lines, while overexpression of miR-139-5p reversed CD44+/CD133+-associated MDR. We also identified NOTCH1, an important protein for stem cell maintenance and function, as a direct target of miR-139-5p, both *in vitro* and in a knockout mouse model. Notch1 expression was upregulated in tumor samples and inversely correlated with expression of miR-139-5p. Silencing *NOTCH1* exerted an effect similar to overexpression of miR-139-5p by inhibiting the CD44+ and CD133+ population and reversing the drug-resistant phenotype. In conclusion, miR-139-5p downregulated NOTCH1 signaling to reverse CD44+/CD133+-associated MDR in colorectal cancer cells. Given this insight into the miRNA regulation of MDR, miR-139-5p could be a promising therapeutic target for colorectal cancer therapy.

## INTRODUCTION

Colorectal cancer (CRC) is the third most common cause of cancer-related death, but treatment of CRC often fails to eradicate all of the tumor cells because the cells have intrinsic or acquired drug resistance [1, 2]. The mechanisms by which cells develop multidrug resistance (MDR) have been extensively studied, yet the cause of MDR is still unknown [3]. Therefore, understanding the mechanism of MDR in CRC cells is crucial for the optimization of current therapeutic techniques.

Cancer stem cells (CSCs) are thought to be key promoters of drug resistance [4–6], particularly to chemotherapeutic drugs [4, 7, 8]. In CRC, CSCs may correlate with MDR [7–9]. CSCs can be defined by their high expression of CD44 [10] and CD133, and this unique phenotype allows the identification of colorectal CSCs as a distinct population from the bulk tumor cells. This CD44^+^/CD133^+^ cell population is thought to initiate and sustain tumor growth, and thus is an obvious target for therapeutic treatment [11]. The CD133^+^/CD44^+^ population, which comprises the cancer initiating cells (CICs) [12], may be the best biomarker for the early detection of CRC [13–15].

As miRNAs may promote or suppress tumorigenesis by binding to various targets, there has been intensive research into miRNAs as cancer biomarkers and treatment targets [16]. We previously reported that miR-139-5p expression was frequently lower in CRC patient samples than in normal colorectal tissues, and correlated significantly with advanced clinical stages [17]. MiR-139-5p has also been validated as tumor suppressor in other cancers, including gastric carcinoma [18], breast carcinoma [19], and hepatocellular carcinoma [20, 21].

In this study, we used oxaliplatin (L-OHP; a platinum anticancer drug for the treatment of CRC) and vincristine (VCR; a broad-spectrum anticancer drug) to establish a drug-resistant cell model, and explored the involvement of CSCs, miR-139-5p, and its potential target (*NOTCH1)* in CRC drug resistance.

## RESULTS

### MiR-139-5p function is associated with MDR

**Figure 1:**
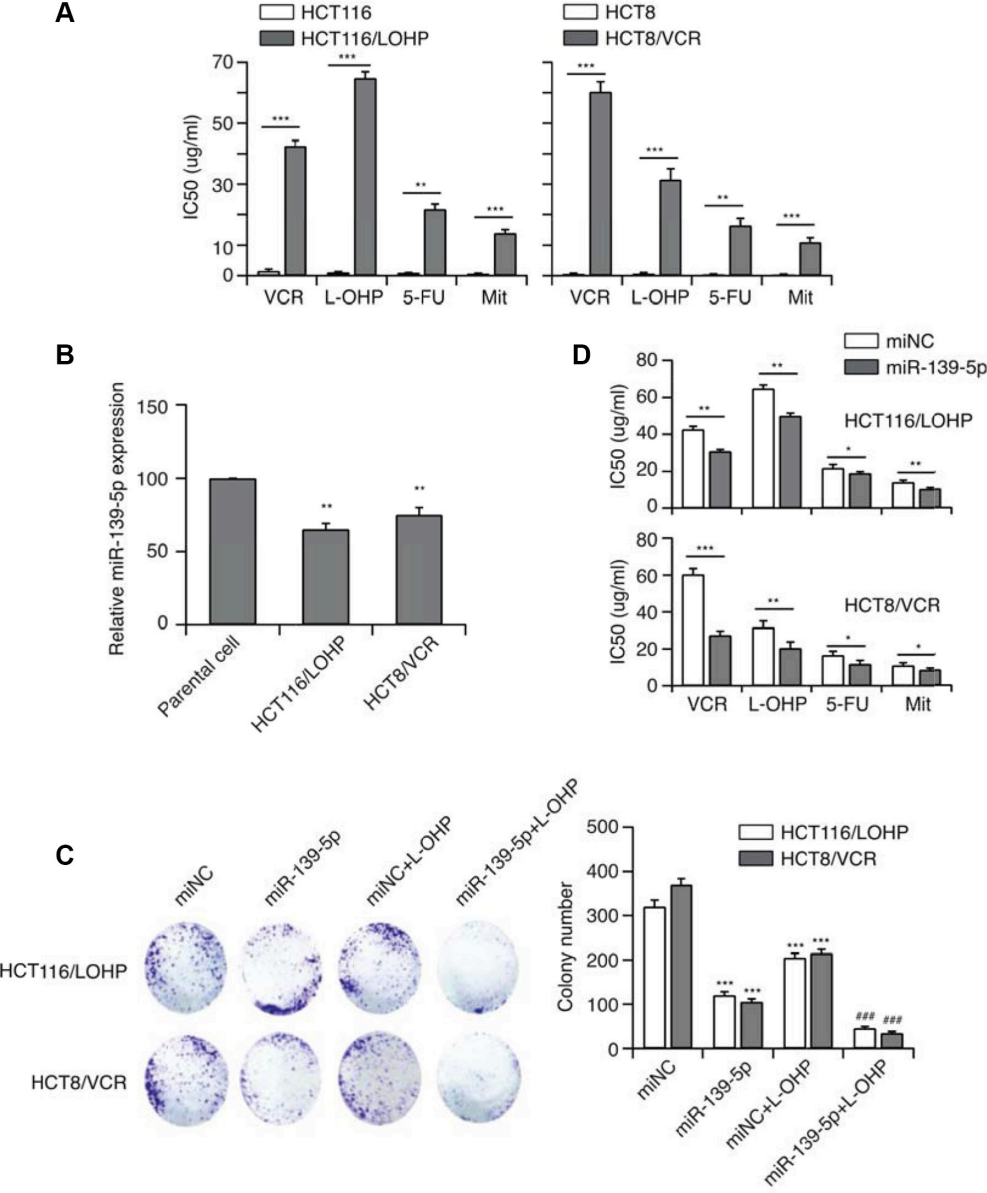
MiR-139-5p function is associated with MDR (**A**) The establishment of MDR cell lines. (**B**) The expression of miR-139-5p in two CRC drug-resistant cell lines. (**C**) The effect of miR-139-5p on the *in vitro* proliferation of both drug-resistant CRC cell lines, and the synergistic effect of miR-139-5p with anticancer drugs (***p < 0.001 miR-139-5p or drug-treated versus control, ^###^*p* < 0.001 miR-139-5p and drug co-treated versus control). (**D**) MiR-139-5p expression significantly enhanced the sensitivity of HCT-116 cells to VCR, L-OHP, 5-FU and Mit, and significantly reduced their IC50 values, based on the CCK-8 assay.

We established L-OHP- or VCR-resistant human CRC cell lines (HCT116/L-OHP and HCT8/VCR, respectively) by repeatedly exposing cells to these drugs. The cells also displayed significant cross-resistance to fluorouracil (5-FU) and mitomycin C (Mit) (Figure 1A).

To evaluate the relationship of miRNA with MDR, we used qRT-PCR to examine the expression of miR-139-5p in drug-resistant cells. MiR-139-5p expression was approximately 30–40% lower (*p* < 0.01) in drug-resistant cancer cells than in the parental stains (Figure 1B). Thus, miR-139-5p expression may inversely correlate with MDR. With this in mind, we next transfected cells with miR-139-5p mimics, and confirmed the re-expression of mature miR-139-5p by qRT-PCR. Intriguingly, increased expression of miR-139-5p suppressed colony formation (Figure 1C). Notably, forced miR-139-5p expression significantly enhanced the sensitivity of HCT116/L-OHP and HCT8/VCR to VCR, L-OHP, 5-FU and Mit, and significantly reduced their half-maximal inhibitory concentration (IC50) (Figure 1D). As miR-139-5p increased the sensitivity of drug-resistant strains to antitumor drugs, miR-139-5p may be able to reverse MDR.

### MDR correlates with CSC properties

**Figure 2:**
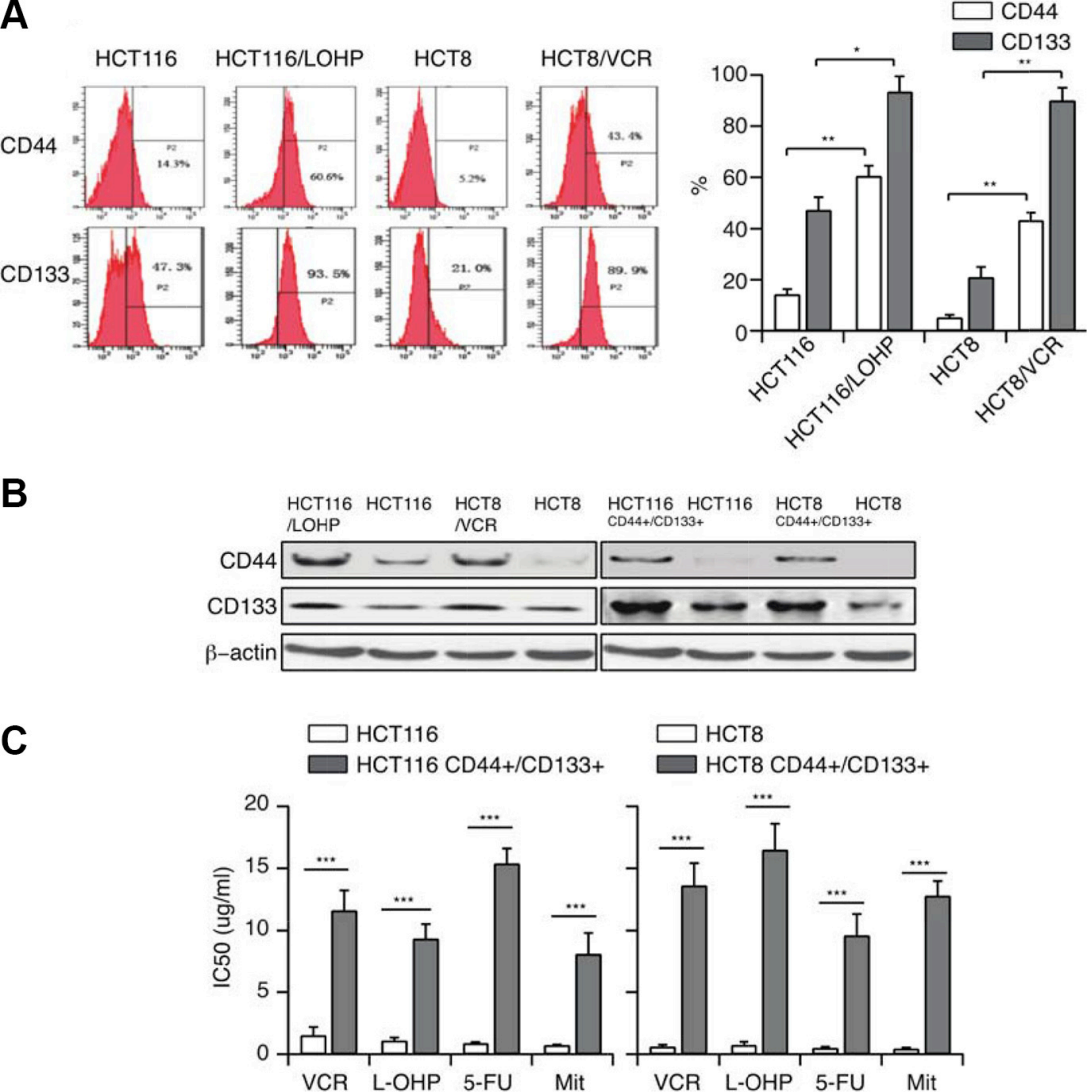
MDR correlates with CSC properties (A, B) CD44 and CD133 expression in HCT116/LOHP and HCT8/VCR cells were determined by flow cytometry and Western blotting (**C**) The IC50 values of VCR, L-OHP, 5-FU and Mit in CD44+/CD133+ cells were determined with a CCK-8 assay.

We speculated that drug-resistant cells may have cancer stem cell-like characteristics, which can be detected by the CSC surface markers CD44 and CD133. Flow cytometry of control and drug-resistant HCT116 and HCT8 cells revealed that the expression of these surface markers increased, especially CD133 (~90%, Figure 2A), with the emergence of the resistant phenotype. This increase in CD44 and CD133 expression demonstrated that a drug-resistance phenotype could correlate with CSC properties. To confirm this hypothesis, we established HCT116 CD44+/CD133+ and HCT8 CD44+/CD133+ cell lines by sorting HCT116 and HCT8 cells. The percentage of CD44+/CD133+ cells in this sorted population could be maintained around 95%. The high levels of CD44 and CD133 in HCT116 CD44+/CD133+ and HCT8 CD44+/CD133+ cells were also confirmed by Western blotting (Figure 2B). We then treated these cells with different anticancer drugs, and found that the IC50 values of VCR, L-OHP, 5-FU or Mit were significantly higher in CD44+/CD133+ cells than in the parental cells (Figure 2C). The above studies illustrated that CD44^+^/CD133^+^ cells have both CSC-like characteristics and MDR capacities, indicating that MDR correlates with CSC properties.

### MiR-139-5p reverses CD44+/CD133+-associated MDR

**Figure 3:**
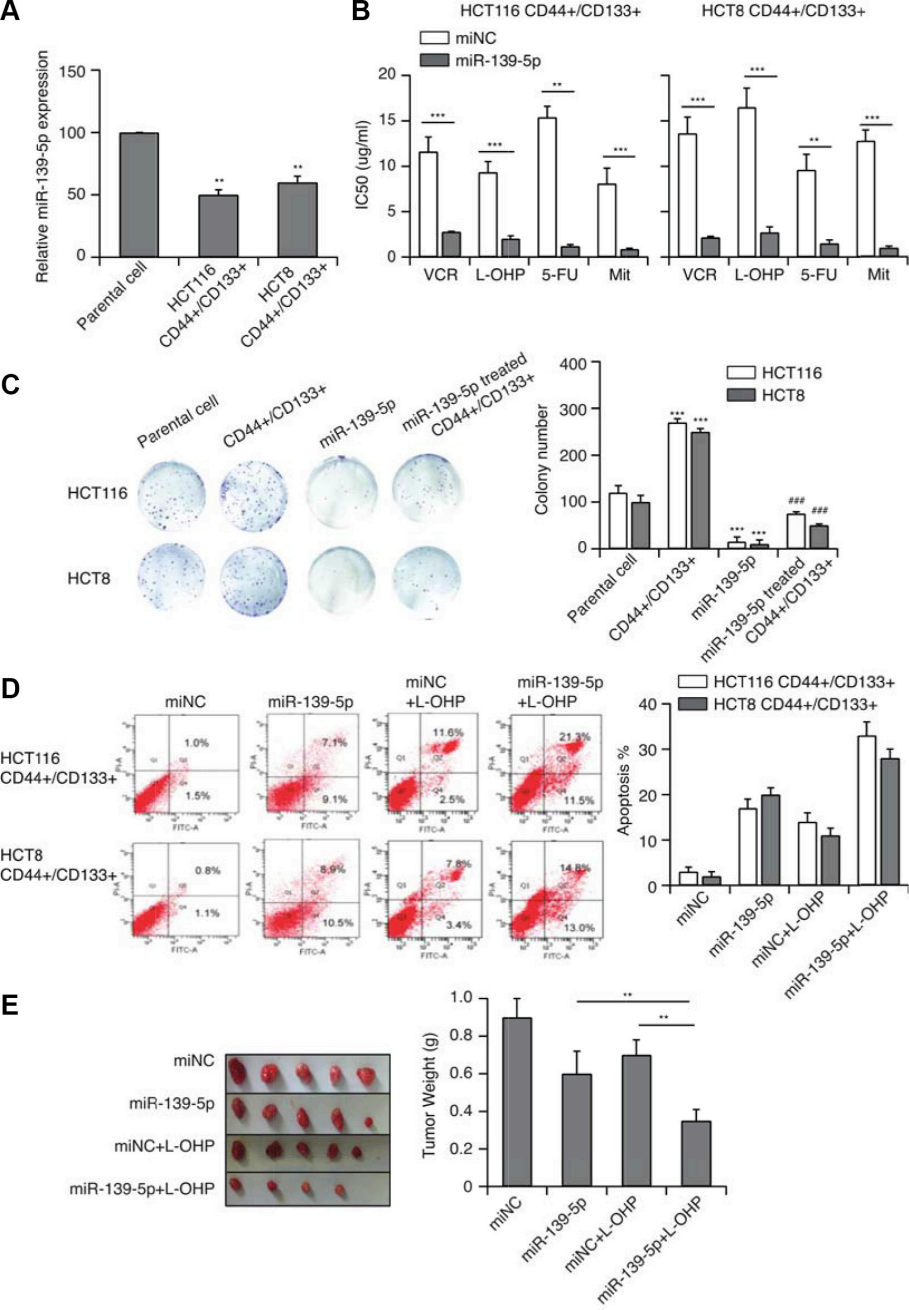
MiR-139-5p reverses CD44+/CD133+-associated MDR (**A**) The expression of miR-139-5p in CD44+/CD133+ cells (**p < 0.01). (**B**) MiR-139-5p expression significantly enhanced the sensitivity of CD44+/CD133+ cells to VCR, L-OHP, 5-FU and Mit, and significantly reduced their IC50 values, based on a CCK-8 assay. (**C**) The effect of miR-139-5p on the *in vitro* proliferation of CD44+/CD133+ cells. (**D**) MiR-139-5p-transfected CD44+/CD133+ cells exhibited enhanced sensitivity towards VCR, L-OHP, 5-FU and Mit, with a greater extent of apoptosis after treatment. (**E**) Tumors derived from the vector- and miR-139-5p-transfected HCT116 CD44+/CD133+ cells were implanted subcutaneously. The weights of the harvested tumors were measured. Each figure is representative of three independent experiments.

We next performed qRT-PCR to evaluate miR-139-5p expression in CD44+/CD133+ cells. MiR-139-5p expression was approximately 50% lower (*p* < 0.01) in CD44+/CD133+ cells than in the parental group (Figure 3A). When we transfected these cells with miR-139-5p mimics and/or treated them with various anticancer drugs, forced miR-139-5p expression significantly enhanced the sensitivity of CD44+/CD133+ cells to VCR, L-OHP, 5-FU and Mit, and significantly reduced their IC50 (Figure 3B). Increased expression of miR-139-5p also suppressed colony formation (Figure 3C). Because the induction of apoptosis is an important indicator of the chemotherapeutic sensitivity of cancer cells, we used flow cytometry to detect the apoptotic capacity of miR-139-5p/control cells in the presence of L-OHP at the IC50. After 24-hour treatment, miR-139-5p-transfected cells displayed greater sensitivity towards these chemotherapeutic agents, as more cells underwent apoptosis after the treatment (Figure 3D).

We also subcutaneously implanted tumors derived from the vector- and miR-139-5p-transfected HCT116 CD44+/CD133+ cells into mice. *In vivo*, CD44^+^/CD133^+^ HCT116 cells had a stronger tumorigenic potential than cells treated with miR-139-5p, L-OHP, or miR-139-5p+L-OHP (Figure 3E). Thus, miR-139-5p increased the sensitivity of CD44^+^/CD133^+^ cells to antitumor drugs, suggesting that miR-139-5p could reverse CD44+/CD133+-associated MDR.

### MiRNA-139-5p directly binds to and functionally suppresses *NOTCH1*

**Figure 4:**
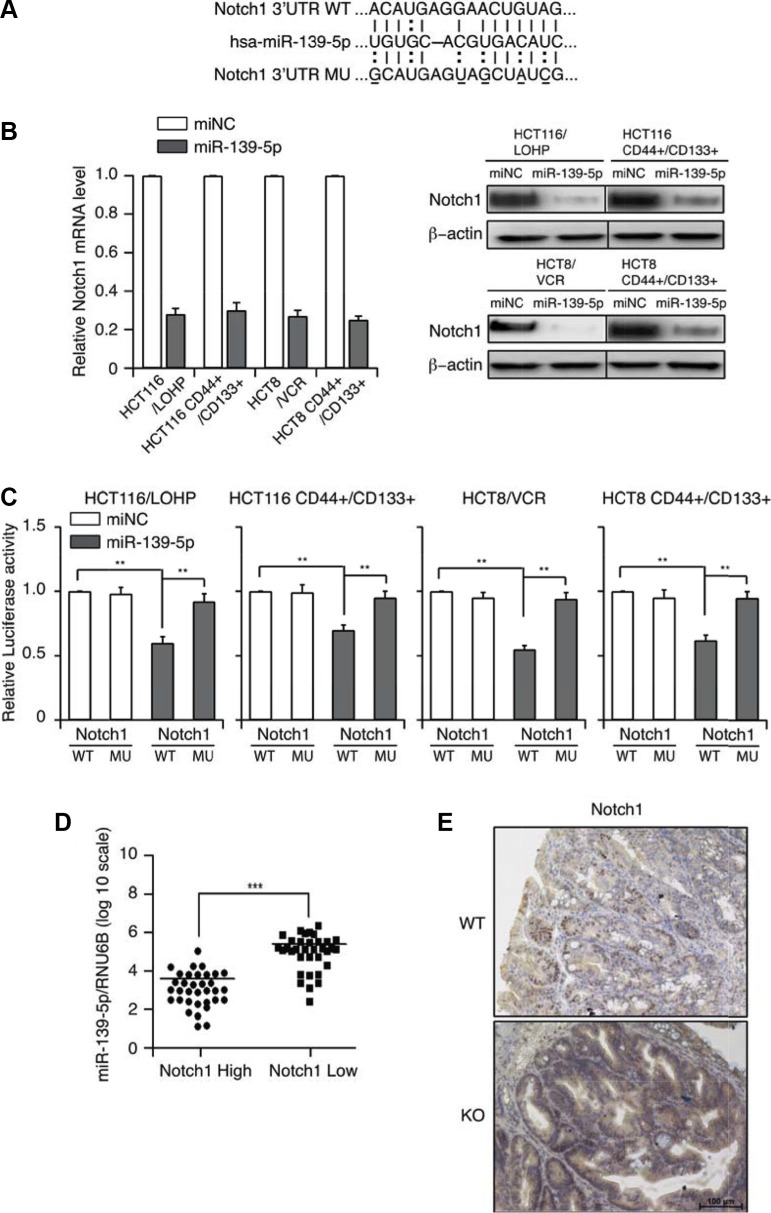
MiR-139-5p directly binds to *NOTCH1* in colon cancer cells (**A**) Schematic representation of putative miR-139-5p binding sites in the 3′-UTR of *NOTCH1* mRNA, and the mutations introduced into the *NOTCH1* 3′-UTR regions. (**B**) *NOTCH1* mRNA and protein expression were determined by qRT-PCR and Western blotting. (**C**) Wild-type (WT) or mutated (MUT) *NOTCH1* reporter constructs were co-transfected with miR-139-5p into drug-resistant or CD44+/CD133+ cells. The relative luciferase activities were measured. (**D**) MiR-139-5p expression correlated inversely with *NOTCH1* expression in CRC samples. (**E**) Representative images of the immunohistochemical examination of Notch1 in mouse tumors from miR-139-5p knockout and WT mice. Each figure is a representative of three independent experiments.

Bioinformatic approaches have identified multiple mRNAs as direct targets of miR-139-5p. Among the predicted targets, we chose to further investigate *NOTCH1* because of its importance in tumorigenesis, tumor progression [22, 23] and stem cell maintenance [24]. MiRNA target searches with Targetscan and Miranda confirmed that *NOTCH1* has a putative miR-139-5p binding site within its 3′-UTR (Figure 4A). To investigate whether miR-139-5p could up- or downregulate *NOTCH1*, we assessed *NOTCH1* mRNA and protein expression in miR-139-5p mimic-transfected cells. MiR-139-5p mimics significantly suppressed *NOTCH1* mRNA and protein expression (Figure 4B). These results suggested that miR-139-5p may downregulate *NOTCH1* expression at both the transcriptional and post-transcriptional levels.

To investigate the possible interaction between miR-139-5p and *NOTCH1*, we introduced mutations into the putative miR-139-5a binding sites in the *NOTCH1* 3′-UTR, generated luciferase reporter constructs with the wild-type (WT) and mutant (MUT) 3′-UTRs of *NOTCH1*, and co-transfected these constructs into CD44^+^/CD133^+^ cells with the miR-139-5a mimics or control vectors. The ectopic expression of miR-139-5a significantly reduced the luciferase activity of the WT *NOTCH1* 3′-UTR, but not of the MUT *NOTCH1* 3′-UTR, in CD44^+^/CD133^+^ cells (Figure 4C).

We further validated the association between miR-139-5p and *NOTCH1* expression in 66 CRC specimens, and found that high miR-139-5p expression was always associated with low *NOTCH1* expression. A Pearson Chi-square test confirmed that miR-139-5p expression inversely correlated with *NOTCH1* expression (*p* < 0.001) (Figure 4D). Importantly, tumors from miR-139-5p knockout mice exhibited stronger Notch1 staining than those from control mice (Figure 4E). These results suggested that *NOTCH1* is indeed a target of miR-139-5p.

### MiR-139-5p reverses CD44+/CD133+-associated MDR, partly by downregulating *NOTCH1*

**Figure 5:**
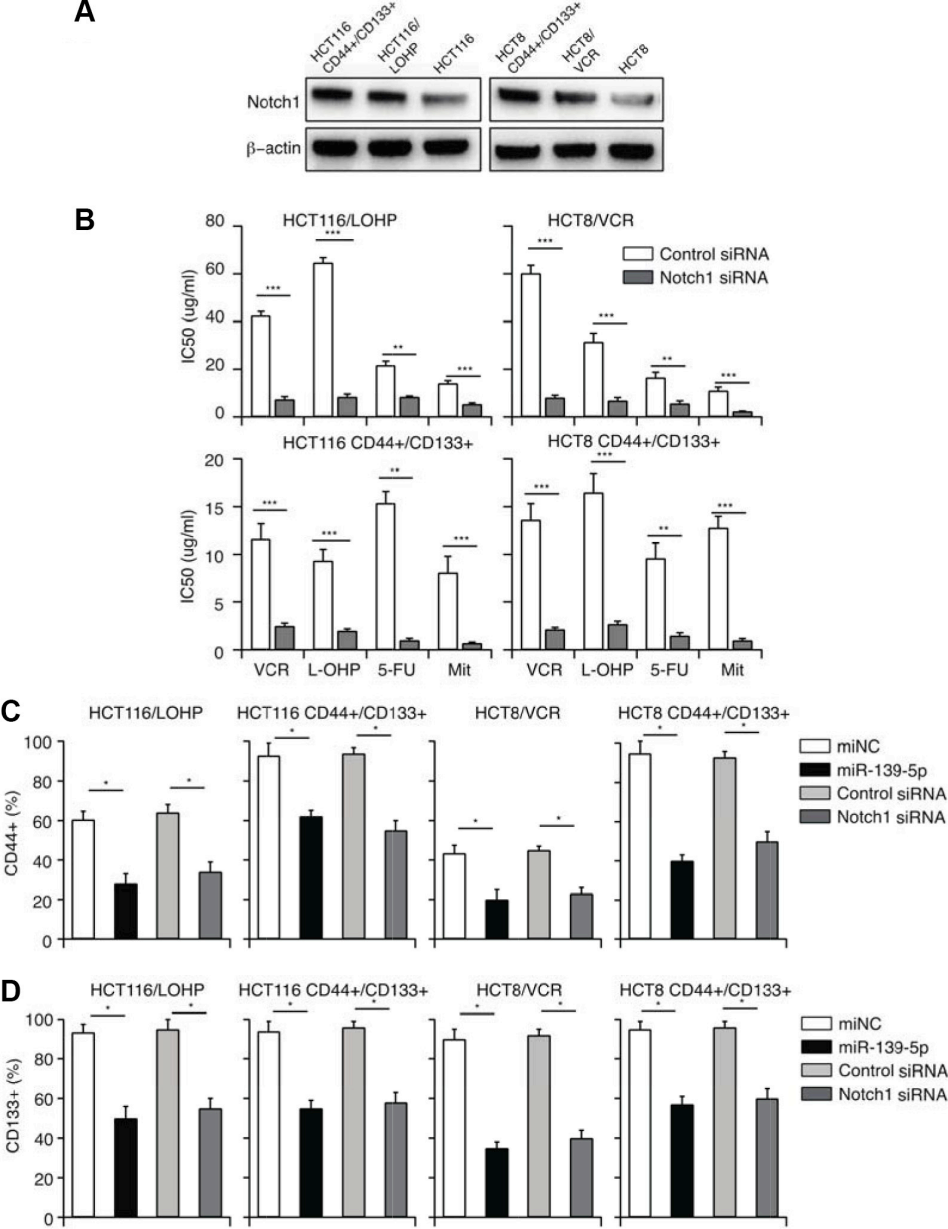
MiR-139-5p reverses CD44+/CD133+-associated MDR, partly by downregulating *NOTCH1* (**A**) The expression of *NOTCH1* in drug-resistant and CD44+/CD133+ cells. (**B**) *NOTCH1* siRNA significantly enhanced the sensitivity of drug-resistant and CD44+/CD133+ cells to VCR, L-OHP, 5-FU and Mit, and significantly reduced their IC50 values, based on a CCK-8 assay. (**C**) The percentage of CD44+ cells was reduced by overexpression of miR-139-5p or knockdown of *NOTCH1*. (**D**) The percentage of CD133+ cells was reduced by overexpression of miR-139-5p or knockdown of *NOTCH1*.

The presence of CSCs is one of the most important reasons for MDR [25–27]. As *NOTCH1* promotes the maintenance of CSCs, we hypothesized that miR-139-5p reverses CD44+/CD133+-associated MDR by downregulating *NOTCH1*. To examine the involvement of miR-139-5p/*NOTCH1* in CD44+/CD133+-associated MDR, we first compared miR-139-5p and *NOTCH1* expression in CD44^+^/CD133^+^ cells and their parental cells. As shown in Figures 3A and 5A, miR-139-5p expression was significantly lower, and NOTCH1 protein expression was greater, in CD44^+^/CD133^+^ cells than in parental cells.

We then transfected miR-139-5p mimics or *NOTCH1* siRNA into drug-resistant or CD44^+^/CD133^+^ cells. A CCK-8 assay demonstrated that *NOTCH1* siRNA treatment reversed CD44+/CD133+-associated MDR, with the same efficiency as miR-139-5p treatment (Figure 5B). These results suggested that miR-139-5p reverses CD44+/CD133+-associated MDR by downregulating *NOTCH1*. To further investigate the efficiency of miR-139-5p mimics and *NOTCH1* siRNA in destroying CSCs, we quantified the number of cells with the CSC markers (CD44 and CD133) by flow cytometry. Seventy-two hours after transfection, the percentage of CD44+ and CD133+ cells had significantly decreased in both the miR-139-5p mimic group and the *NOTCH1* siRNA group (Figure 5C and 5D). Our data confirmed that miR-139-5p reversed the CD44+/CD133+-associated MDR, at least partly, by downregulating *NOTCH1*.

### Overexpression of *NOTCH1* ablates the inhibitory effects of miR-139-5p on MDR in CRC cells

**Figure 6:**
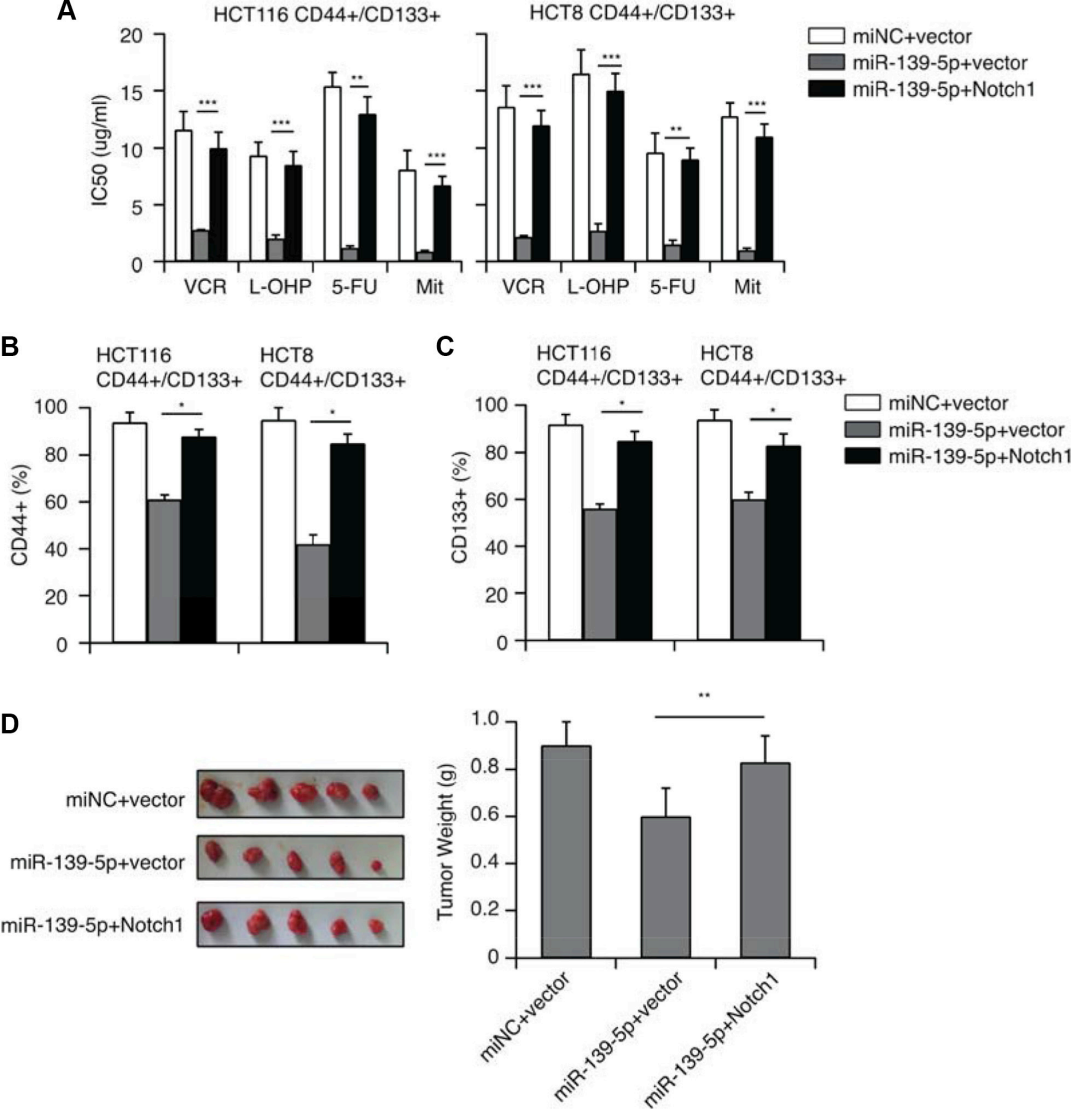
*NOTCH1* restoration counteracts the inhibitory effects of miR-139-5p in CD44+/CD133+ cells (**A**) *NOTCH1* restoration significantly increased the IC50 values reduced by miR-139-5p. (**B**) The percentage of CD44+ cells reduced by miR-139-5p was partly reversed by *NOTCH1* restoration. (**C**) The percentage of CD133+ cells reduced by miR-139-5p was partly reversed by *NOTCH1* restoration. (**D**) Tumors derived from HCT116 CD44+/CD133+ cells transfected with the vector, miR-139-5p or miR-139-5p plus *NOTCH1* were implanted subcutaneously. The weights of the harvested tumors were measured.

To further validate that miR-139-5p inhibited the CD44+/CD133+-associated MDR by downregulating *NOTCH1*, we transfected miR-139-5p mimics into CD44+/CD133+ cells with a pcDNA3.1 vector, or with a pcDNA3.1-NOTCH1 plasmid encoding the full-length coding sequence of the *NOTCH1* intracellular domain without the 3′-UTR. CCK-8 assays revealed that overexpression of *NOTCH1* reversed the inhibitory effects of miR-139-5p on CRC cell drug resistance (Figure 6A). Flow cytometry analysis indicated that the ectopic expression of *NOTCH1* counteracted the inhibition of the CD44+ and CD133+ population resulting from miR-139-5p overexpression (Figure 6B and 6C). An *in vivo* study also demonstrated that overexpression of *NOTCH1* ablated the inhibitory effects of miR-139-5p on tumor growth (Figure 6D). All these results provided further evidence that *NOTCH1* expression is inhibited by miR-139-5p, and that the downregulation of miR-139-5p is a key promoter of CD44+/CD133+-associated colorectal drug resistance because it potentiates *NOTCH1* expression.

## DISCUSSION

Even when CRC is diagnosed early and treated with advanced surgical strategies, a considerable proportion of CRC patients develop recurrence or metastasis within 5 years of surgical treatment [28]. In many cases of tumor recurrence, the cancer cells are resistant to conventional chemotherapy. CSCs are postulated to be important promoters of MDR [29]. Although medicines such as 5-FU and L-OHP are available for CRC treatment, drug resistance limits their clinical application.

Considering the clinical significance of drug resistance and the ineffectiveness of chemotherapy in removing CSCs, we evaluated the correlation of chemo-resistance with the CSC phenotype. Consistent with reports that chemo-resistant CRC cells are enriched for CSCs, we discovered that the expression of CD44 and CD133 increased in drug-resistant cells.

*NOTCH1*, as the target gene of miR-139-5p, has key functions in miR-139-5p- induced cell migration, invasion [30], apoptosis and drug re-sensitization [31]. We further confirmed that *NOTCH1* is a direct target of miR-139-5p in CRC cells, and showed that miR-139-5p suppresses tumor growth by downregulating *NOTCH1* expression. Notch signaling has also been suggested to determine cell fate, for instance, by promoting the self-renewal of stem cells and cell differentiation [32]. Furthermore, the Notch pathway is a key promoter of cell survival and cell proliferation. Dysregulated expression of Notch receptors has been found in different neoplastic lesions, indicating that Notch may act as an oncogene [33]. High expression of *NOTCH1* is related to poorer overall survival in patients. *NOTCH1* is also regarded as a diagnostic and prognostic biomarker in clinical diagnosis and treatment.

Our study first found that miR-139-5p reversed CD44+/CD133+-associated MDR, partly by downregulating *NOTCH1 in vitro*. Compared with the parental cells, higher expression of *NOTCH1* was found in drug-resistant or CD44^+^/CD133^+^ cells, which expressed lower levels of miR-139-5p (Figure 5A). Overexpression of miR-139-5p increased drug sensitivity and suppressed the CD44+/CD133+ population in drug-resistant and CD44+/CD133+ cells by downregulating *NOTCH1*. In CRC samples and miR139-5p knockout mice, miR-139-5p expression inversely correlated with NOTCH1 expression. This suggested that miR-139-5p downregulation promotes CD44+/CD133+-associated colorectal drug resistance by disinhibiting *NOTCH1*.

This deeper study of miR-139-5p has expanded our understanding of CRC. MiR-139-5p could be a clinically feasible target for drug design, either if a single stimulatory agent is developed specifically for miR-139-5p, or if treatments targeting miR-139-5p are combined with classical chemotherapeutic drugs. Such therapies could enhance chemosensitivity by directly or indirectly reducing the expression of resistance-associated proteins, as well as by inhibiting the cell cycle and promoting apoptosis in CRC cells.

Biological characterization of colorectal CSCs may help clinicians reduce tumor recurrence and improve the diagnosis and treatment of CRC. Since the first description of CSCs, significant advances have been made in our understanding of their function in hematopoietic and solid tumors - they are now known to contribute significantly to tumorigenesis and the recurrence of malignancies by causing drug resistance and promoting cancer progression. Therefore, suppressing CSCs is an efficient therapeutic method for treating various cancers. In this study, we investigated the function of miRNA in CSCs, and found that the downregulation of miR-139-5p caused drug resistance and cancer progression. Thus, our data are applicable to the development of novel therapeutic strategies to stimulate or inhibit miRNAs in CSCs to better treat cancer patients.

In conclusion, the present study demonstrated that in CD44^+^/CD133^+^ CSC-like cells, miR-139-5p could reverse MDR by downregulating *NOTCH1*. These findings suggest the potential feasibility of miR-139-5p as a biomarker and novel anti-cancer drug target, and provide a full-scale theoretical basis and new strategy for CRC diagnosis, targeted therapy and prognosis.

## MATERIALS AND METHODS

### Cell culture and plasmids

Human CRC cell lines (HCT116, HCT8) were purchased from the Cell Bank of the Type Culture Collection of the Chinese Academy of Sciences (Shanghai, China). All cells were cultured in a monolayer at 37°C in a humidified atmosphere of 5% CO_2_ and 95% air in Rosewell Park Memorial Institute (RPMI) 1640 medium supplemented with 10% fetal bovine serum. The plasmid used in the experiments was designed as previously reported [17]. Transfection of the cells was performed with Lipofectamine^™^ 2000 according to the manufacturer's protocol.

### Patient samples

Human CRC samples and corresponding nontumorous colorectal samples were collected at the time of surgical resection from consenting patients, with ethical approval from the research ethics committees of Putuo Hospital. Samples were immediately snap frozen in liquid nitrogen and stored at −80°C. The experiments were conducted with the understanding and written consent of each subject, and the study methodologies accorded with the standards set by the Declaration of Helsinki.

### RNA purification and qRT-PCR analyses

Total RNA was extracted with Trizol (Invitrogen Corporation, Carlsbad, California, USA) according to the manufacturer's instructions. Reverse transcription was performed with a One Step PrimeScript miRNA cDNA Synthesis Kit (Takara Bio Inc., Dalian, China). qRT-PCR was performed with a miR-139-5p MiRNA TaqMan Assay (Applied Biosystems) on an iCycler thermal cycler (Bio-Rad, Hercules, USA). U6 RNA was used as a miRNA internal control. The primer used for qRT-PCR for miR-139-5p was: 5′-TCT ACA GTG CAC GTG TCT CCA G-3′.

### Proliferation assays

The proliferation of cells was evaluated with the CCK-8 assay (Kumamoto, Japan) according to the manufacturer's instructions. Cells were plated in 96-well plates at a density of 2 × 10^3^ cells/well. Cells were transfected with miRNA mimics 24 h later, and were cultured for 48 h before the addition of 10 mL of CCK-8 to each well. After another 4 h of incubation at 37°C, the optical density of each well was examined with a Thermomax Microplate Reader at 450 nm. Each experiment was performed three times.

### Colony formation assay

Cells (0.5 × 10^3^) were seeded into a six-well plate and cultured in a humidified incubator at 37°C for 14 days. All samples were washed with PBS, fixed in 75% ethanol, stained with 0.1% crystal violet, and counted.

### Luciferase assay to validate predicted binding sites

Predicted target sites of miR-139-5p in *NOTCH1* were cloned into the Xho I and Not I sites of the psiCheck2 Luciferase vector, as were the mutated miR-139-5p target sites. Cells were co-transfected with 50 ng of psiCheck2-NOTCH1-WT or psiCheck2-NOTCH1-Mut constructs and miR-139-5p mimics or negative control mimics. Luciferase activity was measured 48 h post-transfection with a dual luciferase reporter assay system (Promega Corporation, WI, USA). Firefly luciferase activity was normalized to renilla luciferase activity in each transfected well. Three independent experiments were undertaken in triplicate and independently repeated at least twice.

### Western blot analysis

Cellular proteins were extracted and separated on SDS/PAGE gels, and Western blot analyses were performed according to standard procedures, as previously described [34]. β-actin was used as a loading control.

### Flow cytometry

For apoptosis, an Annexin V-FITC apoptosis detection kit (Invitrogen, USA) was used according to the manufacturer's instructions. For cell sorting, after non-specific binding was excluded, approximately 1×10^6^ cells were labeled with conjugated anti-human CD133-PE (eBioscience, USA) and anti-human CD44-FITC (eBioscience, USA). Cells were resuspended in PBS buffer with 2% FBS and analyzed by flow cytometry. Cell sorting was conducted under sterile conditions, and the sorted cells were cultured as described above.

For cell cycle analysis, the harvested cells were suspended in chilled PBS, fixed with cold 70% ethanol and incubated at 4°C for 10 minutes. Then, cells were incubated with 50 μL RNase for 30 min at 37°C. Finally, cells were incubated with propidium iodide for 10 min in the dark at 4°C. The cell cycle distribution was then examined by flow cytometry.

### Subcutaneous xenografts

For subcutaneous xenografts, the experiment was performed as previously described [17].

### Mouse model

MiR-139-5p knockout mice and littermate controls, all on the C57BL/6 background, were originally purchased from the Genetically Modified Animal Center (East China Normal University at Shanghai). The miR-139-5p gene was knocked out by CRISPR/Cas9 in the whole body as described previously [35]. MiR-139-5p knockout mice were then backcrossed onto the C57BL/6 background for 10 generations. All mice were 6–8 weeks old and bred in-house to generate comparable groups.

Colitis-associated tumorigenesis was induced according to a previously reported protocol [36]. Briefly, mice were injected intraperitoneally with 10 mg/kg azoxymethane (Sigma–Aldrich), and were treated 5 days later with 2.5% dextran sulfate sodium in their drinking water for 7 days, followed by 14 days of regular water. After two additional cycles of this treatment, the mice were sacrificed at the end of the DSS cycle. Body weights were recorded daily. Mice were sacrificed at the indicated time intervals, and the numbers and sizes of tumors were measured in a blinded fashion.

### Histological analysis

Paraffin-embedded colorectal tissues were longitudinally cut into 4-m sections and stained with hematoxylin and eosin solution. Immunohistochemical staining was performed as described previously [37]. A *NOTCH1* rabbit anti-human antibody was used at a dilution of 1:100 (Epitomics, USA); PBS was used as a negative control. Every section was evaluated and scored independently by two pathologists. A semi-quantitative scoring system was used in this trial [37, 38].

### Statistical analysis

All values were expressed as the mean ± SD. Student's *t*-test was performed with GraphPad software to estimate the significance of differences between groups. Statistical significance is indicated as *p < 0.05, **p < 0.01, and ***p < 0.001.
